# Gut microbiota and its metabolic products in acute respiratory distress syndrome

**DOI:** 10.3389/fimmu.2024.1330021

**Published:** 2024-02-16

**Authors:** Dong-Wei Zhang, Jia-Li Lu, Bi-Ying Dong, Meng-Ying Fang, Xia Xiong, Xue-Jun Qin, Xian-Ming Fan

**Affiliations:** ^1^ Department of Respiratory and Critical Care Medicine, The Affiliated Hospital of Southwest Medical University, Luzhou, Sichuan, China; ^2^ Inflammation & Allergic Diseases Research Unit, The Affiliated Hospital of Southwest Medical University, Luzhou, Sichuan, China; ^3^ Department of Respiratory and Critical Care Medicine, Liuzhou People’s Hospital, Guangxi Medical University, Liuzhou, Guangxi, China; ^4^ Key Laboratory of Diagnosis, Treatment and Research of Asthma and Chronic Obstructive Pulmonary Disease, Liuzhou, Guangxi, China; ^5^ Department of Dermatology, The Affiliated Hospital, Southwest Medical University, Luzhou, Sichuan, China

**Keywords:** acute respiratory distress syndrome (ARDS), gut microbiota, metabolic products, gut-lung, axis

## Abstract

The prevalence rate of acute respiratory distress syndrome (ARDS) is estimated at approximately 10% in critically ill patients worldwide, with the mortality rate ranging from 17% to 39%. Currently, ARDS mortality is usually higher in patients with COVID-19, giving another challenge for ARDS treatment. However, the treatment efficacy for ARDS is far from satisfactory. The relationship between the gut microbiota and ARDS has been substantiated by relevant scientific studies. ARDS not only changes the distribution of gut microbiota, but also influences intestinal mucosal barrier through the alteration of gut microbiota. The modulation of gut microbiota can impact the onset and progression of ARDS by triggering dysfunctions in inflammatory response and immune cells, oxidative stress, cell apoptosis, autophagy, pyroptosis, and ferroptosis mechanisms. Meanwhile, ARDS may also influence the distribution of metabolic products of gut microbiota. In this review, we focus on the impact of ARDS on gut microbiota and how the alteration of gut microbiota further influences the immune function, cellular functions and related signaling pathways during ARDS. The roles of gut microbiota-derived metabolites in the development and occurrence of ARDS are also discussed.

## 1 Introduction

Acute respiratory distress syndrome (ARDS) is characterized by a severe inflammatory response, triggered by either primary or secondary acute injury to the lungs. This injury leads to damage to the alveolar wall and subsequent pulmonary congestion, culminating in gas exchange dysfunction (1). The clinical manifestations of ARDS typically include hypoxemia, respiratory distress, and pulmonary rales (2). Commonly associated conditions with ARDS encompass pneumonia, serious trauma, aspiration of gastric contents, or sepsis. Globally, the prevalence rate of ARDS is estimated to be around 10% among critically ill patients (3).

In intensive care unit (ICU) patients, the prevalence of ARDS exceeds 3%, and the incidence rates demonstrate a substantial variation, with differences exceeding 400% between different regions (4, 5). In USA, the incidence rate of ARDS elevated from 180.7/100,000 (in 2006) to 193.4/100,000 (in 2014) (6). A European study involving 3,504 hospitalized patients reported an incidence of ARDS at 32 cases per 100,000 individuals annually (7). In China, another investigation identified 672 out of 18,793 ICU patients as meeting the Berlin ARDS criteria (8). Additionally, the prevalence of ARDS is estimated at approximately 3% among pediatric intensive care unit patients (5). The mortality of ARDS is from 17% and 39% in different studies (9, 10). Presently, high mortality rate of ARDS is usually observed in COVID-19 patients, posing an additional challenge to the treatment of ARDS (11).

The management of ARDS encompasses early prevention, supportive care, pharmacological interventions, and mechanical ventilation (12). Given the substantial morbidity and mortality rates attributed to ARDS, prioritizing preventive strategies and optimized treatment measures are crucial. Observational studies have indicated that antiplatelet therapies, including aspirin, could be advantageous in averting ARDS among high-risk populations (13). In a randomized, double-blind, placebo-controlled trial, inhaled administration of salbutamol for 7 days did not elicit statistically significant differences in measured biomarkers of lung injury or inflammation compared to placebo, suggesting no detectable attenuation of the pathophysiological mechanisms underlying ARDS (14). Another investigation revealed that tracheotomy might extend survival in individuals diagnosed with ARDS (15).

The current therapeutic modalities for ARDS have proven suboptimal in addressing the clinical challenges associated with this condition. Given this unmet need, it is crucial to delve into the underlying pathogenesis and pathophysiological mechanisms of ARDS to identify innovative therapeutic targets. Emerging evidence underscores the integral role of gut microbiota in maintaining physiological homeostasis, modulating immune responses, and facilitating nutrient synthesis. Notably, a growing body of literature suggests that gut microbiota exerts a modulatory effect on the onset and progression of respiratory diseases through its influence on immune responses, oxidative stress, and inflammatory markers. Therefore, a comprehensive understanding of the mechanistic involvement of gut microbiota in ARDS and related respiratory conditions could pave the way for the development of targeted therapeutic interventions.

## 2 Lung microbiota, gut microbiota and the gut-lung axis

The lung microbiota plays a crucial regulatory role in lung and systemic immune modulation. The lung microbiota influences the development and function of immune cells such as macrophages, dendritic cells, etc., thereby impacting pulmonary and overall inflammatory responses (16). Lira et al. demonstrated that the lung microbiota plays a pivotal role in regulating immune responses, specifically influencing the delicate balance between pro-inflammatory and anti-inflammatory signals within the respiratory system (17). Khatiwada et al. confirmed that in the context of COVID-19, the lung microbiota can impact the risk and outcomes of diseases by activating both innate and adaptive immune responses (18). Furthermore, research has elucidated that the lung microbiota interacts with pattern recognition receptors on immune cells through the production of microbial molecules, influencing the host’s immune response (19). Studies have found an increase in the relative abundance of *Enterobacteriaceae* and *Pasteurellaceae* in the lung microbiome of ARDS samples, highlighting a potential shift in microbial composition (20). Dysbiosis refers to the disruption of the balance between beneficial and harmful microbes, with a relative increase or decrease in certain microbial populations. When the composition of the lung microbiota is disrupted, it may lead to immune imbalances, resulting in excessive inflammation and subsequent tissue damage (21).

In addition to the lung microbiota, the gut microbiota and its metabolites also play a regulatory role in lung immune function and inflammatory responses. Gut microbiota refers to the diverse group of microorganisms that reside in the human digestive tract, including bacteria, fungi, and viruses. These microorganisms play an important role in maintaining physiological balance, enhancing immune function, and synthesizing some essential nutrients in the human body (22, 23). In the present contexts, there are significant researches scrutiny directed towards elucidating the intricate interplays between the gut microbiota and diverse physiological systems, encompassing: gut -brain axis (24), gut -liver axis (25), gut -heart axis (26), and gut -skin axis (27) are currently hot topics in research. In this context, research on the gut-lung axis is also growing, aiming to elucidate the impact of gut microbiota and their metabolites on respiratory system diseases.

The gut-lung axis (28) refers to the anatomical independence of the gut and lungs, but the existence of complex interactions between them in terms of physiology and pathology. These interactions involve not only the host-microbe relationship but also the interplay between different microbial communities. The gut-lung axis functions through local regulation and distal effects, affecting the progression of respiratory diseases by modulating immune and inflammatory responses.

The balance of lung and gut microbiota, as well as the metabolic products and the barrier function of the gut microbiota, are essential components of overall health. When diseases occur, it can result in dysbiosis of the lung and gut microbiota, decreased production of beneficial gut microbiota metabolites, leading to impaired gut barrier function and increased intestinal permeability. This allows bacterial cell wall components such as lipopolysaccharide (LPS) and pathogen-associated molecular patterns, or even whole bacteria, to enter the bloodstream or lymphatic circulation, triggering systemic inflammation (29), and accelerating the progression of respiratory diseases (30). Pu et al. uncovered that specific bacteria within the gut microbiota stimulate the migration of group 2 innate lymphoid cells from the intestinal region to the lungs, contributing to host defense by regulating the cytokine IL-33 (31). In Ahlawat and Sharma’s study, they explored the role of the gut-lung axis in immunological coordination during SARS-CoV-2 infection, emphasizing the widespread distribution of the ACE2 receptor in both the gut and lungs. This research highlights how such interconnectivity affects the enteric microbiota and extrapulmonary symptoms in patients with COVID-19 (32). Oliveira and colleagues suggested that gut dysbiosis contributes to increased inflammation in both the gut and lungs or diminishes the capacity for anti-inflammatory processes, which plays a role in the immune response to SARS-CoV-2, potentially being either beneficial or detrimental (33). Yang et al. constructed a model of pulmonary infection and gut dysbiosis in 6-week-old mice, establishing a “gut-lung” communication axis through spleen and blood analyses. They demonstrated that gut dysbiosis disrupts the balance between Th1 and Th2 cells, exacerbating lung injury during infection. Furthermore, dysbiosis can have enduring impacts on the function of alveolar macrophages, which are vital in combating pulmonary infections (34). Similarly, Zhou et al. confirmed that dysbiosis of the gut microbiota exacerbates tracheal injury through the TLR4 signaling pathway, resulting in increased expression of inflammatory factors (34). It has been reported that SCFAs can effectively attenuate LPS-induced ARDS in rats by enhancing intestinal barrier function, modulating signaling pathways such as MAPK and NF-κB, leading to a decrease in the release of inflammatory factors and an increase in the production of anti-inflammatory factors, thus having a modulating effect on immunity and inflammation (35).

Under the coordinated action of the gut-lung axis, the balance of lung microbiota and gut microbiota, the metabolites of gut microbiota, and the intestinal barrier function are indispensable components for the health of the body and the maintenance of the lungs to perform their normal physiological functions. In the context of respiratory diseases, dysbiosis of the lung microbiota can trigger systemic inflammatory responses, thereby adversely affecting the composition of the gut microbiota. Conversely, dysbiosis of the gut microbiota can further disrupt immune system balance, consequently affecting the function and composition of the lung microbiota (36–39). Therefore, a comprehensive understanding and exploration of the regulatory mechanisms of the gut-lung axis in ARDS is expected to provide new therapeutic strategies and drug targets for the prevention and treatment of ARDS. This not only facilitates the precise and efficient treatment of ARDS but also alleviates the economic burden on both patients and society, ultimately enhancing the overall quality of life for affected individuals.

## 3 Gut microbiota in ARDS

**Figure 1:**
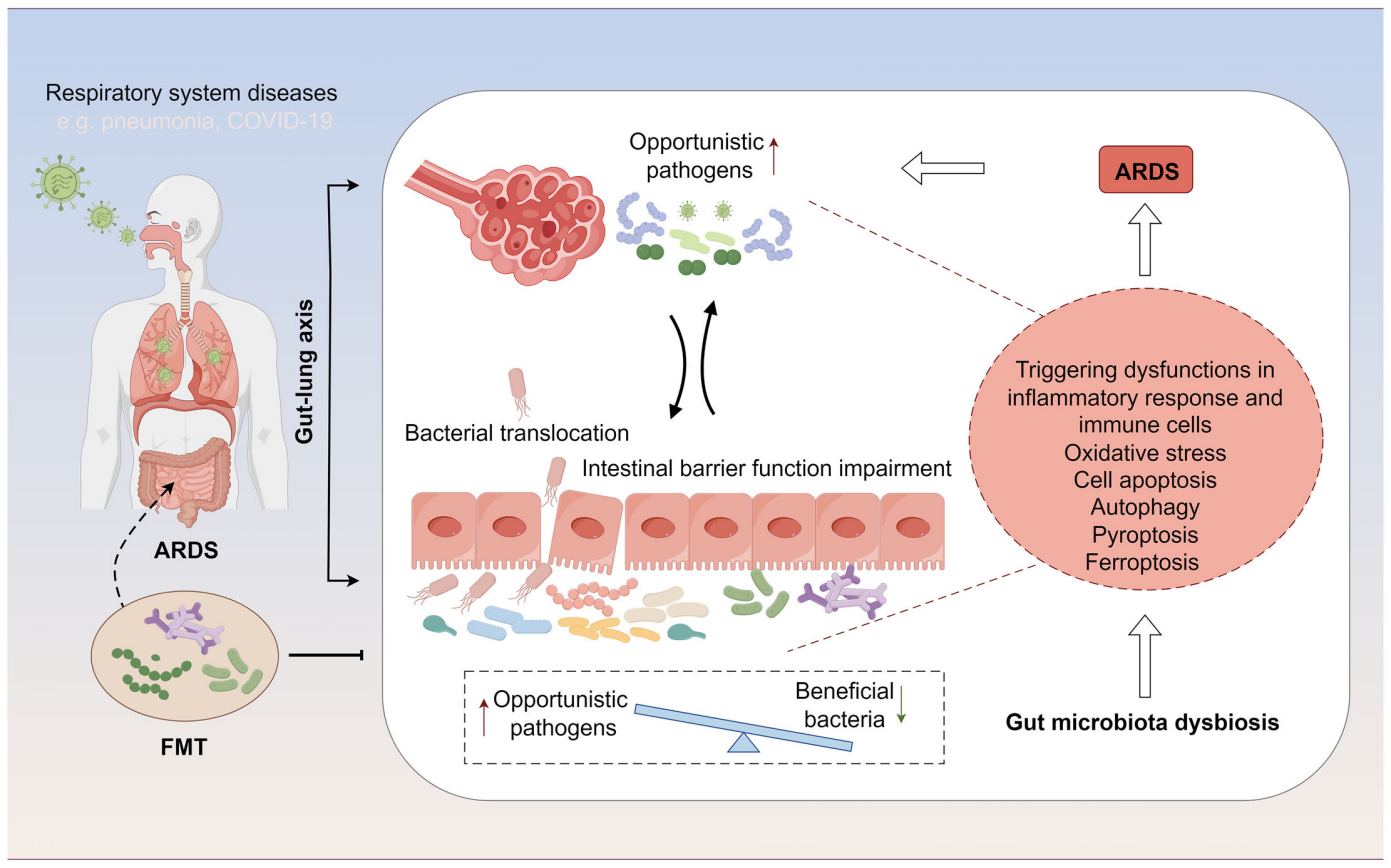
The gut-lung axis represents a bidirectional regulatory pathway between the gut microbiome and ARDS. On one hand, ARDS alters the distribution of the gut microbiome and affects the intestinal mucosal barrier through changes in the microbial composition. On the other hand, modulation of the gut microbiome can influence the development and progression of ARDS by further impacting immune functions, cellular functions, and related signaling pathways during ARDS.

In patients with ARDS, gut microbiota dysbiosis manifests as a reduction in beneficial symbiotic bacteria and an increase in opportunistic pathogens. Specifically, there is a decrease in beneficial bacterial populations such as *Faecalibacterium prausnitzii*, *Bacteroides*, and *Bifidobacterium*, which play a crucial role in maintaining the intestinal mucosal barrier and modulating local and systemic immune responses (40, 41). Concurrently, there is an increase in the abundance of pathogenic or conditionally pathogenic microbes, such as certain *Enterobacteriaceae* members, *Clostridium difficile*, and some *Streptococcus* species, which may lead to intestinal inflammation, impaired intestinal barrier function, and thereby affect systemic inflammatory status and immune responses (42, 43) (**Table 1**). Moreover, the altered gut microbiota landscape in ARDS patients plays a pivotal role in influencing cellular processes and recovery from injury, impacting oxidative stress responses, inflammatory cascades, and various types of cell death, such as apoptosis, pyroptosis, autophagy, and ferroptosis(**Figure 1**). Understanding these alterations and their reciprocal influence on cellular functions during ARDS progression is crucial for identifying novel therapeutic targets and strategies.

**Table 1:** Gut microbiota in ARDS.

Literature Number	Type	Belongs to Phylum or Genus	Microbial Name	Changes in ARDS
(40, 41, 44) (45) (46, 47) (48) (47)	Beneficial Pathogenic	Genus *Lactobacillus* Genus *Eubacterium* Genus *Clostridium* Genus *Clostridium* Phylum *Proteobacteria*	*Lactobacillus*, *Bifidobacterium*, *Faecalibacterium prausnitzii* *Eubacterium rectale* *Clostridium butyricum, Clostridium leptum* *Clostridium hathewayi*, *Clostridium ramosum* *Proteobacteria, Enterobacteriaceae, Shigella, Klebsiella Pneumoniae*	Downregulated Downregulated Downregulated Upregulated Upregulated

### 3.1 The alteration of gut microbiota in ARDS

ARDS may exert influence on various gut microbiota, and these differences may be correlated with the distinct primary diseases that lead to ARDS. The study indicated that in ARDS caused by SARS-CoV-2 infection, Clostridia such as *Clostridium hathewayi* and *Clostridium ramosum* are upregulated in the intestinal tract of COVID-19 patients (49). A study by Hu et al. revealed specific alterations in the function of gut microbiota in acute pancreatitis patients who developed ARDS. In these AP-ARDS patients, there was a higher abundance of *Proteobacteria*, *Enterobacteriaceae*, *Shigella*, and *Klebsiella Pneumoniae*, but a lower abundance of *Bifidobacterium*. These findings suggest that changes in gut microbiota might serve as predictive indicators for the onset of ARDS (47). Panzer et al. demonstrated that the structure of the respiratory microbiota following severe blunt trauma was associated with the development and onset of ARDS (48). Zuo et al. demonstrated that the fecal microbiota in COVID-19 patients with ARDS underwent sustained changes during hospitalization and was related with disease severity compared to controls (48). Chen et al. provided evidence suggesting that a pre-existing diverse and rich gut microbiota may be advantageous in preventing severe COVID-19 and ARDS (50). Additionally, the supplementation with symbiotic microbial metabolites could potentially enhance the therapeutic approach to severe COVID-19 (50). The research findings of Kim et al. (45) and Tang et al. (46) further support the previously mentioned studies. They demonstrate that specific organisms, including *Faecalibacterium prausnitzii*, *Clostridium butyricum*, *Clostridium leptum*, and *Eubacterium rectale*, show altered abundance in COVID-19 patients with ARDS. These organisms may potentially serve as valuable diagnostic biomarkers.

### 3.2 The influence of gut microbiota on intestinal mucosal barrier in ARDS

The intestinal mucosal barrier is essential for maintaining gut health, balancing microbiota through antimicrobial peptides, and the combined action of the mucous layer and cell junctions (51). On the contrary, the intestinal mucosal barrier can also be regulated or attacked by changes in gut microbiota, increasing mucosal permeability and leading to the activation of the immune system and inflammatory response (52). A compromised barrier leads to intestinal inflammation and heightened exposure to external pathogens, while gut microbiota also affect bodily functions via mechanisms like inflammation and oxidative stress (53).

In various diseases, gut microbiota affects the intestinal barrier through cellular functions, inflammation, and oxidative stress. Cocaine use in mice upregulates NF-κB and IL-1β, altering gut microbiota and impairing intestinal permeability via the MAPK/ERK1/2 pathway (54). *Scutellaria baicalensis* (SC) helps diabetic rats by preserving the intestinal barrier, reducing inflammation via TLR-4/TRIF and TNFR-1/NF-κB pathways, and altering gut microbiota, affecting several bacterial genera (55). The study conducted by Yu et al. revealed that the sulfated derivative of *Cyclocarya paliurus* polysaccharide (SCP3) significantly modified the composition of the gut microbiota, restoring the intestinal barrier by enhancing oxidative stress resistance and upregulating tight junction proteins (56). Ge et al. showed that oxidative products in mice lead to weight gain, disruption of the intestinal barrier, and induction of inflammatory and oxidative stress, consequently altering the gut microbiota and impacting various bacteria, such as *Akkermansia* and *Lactobacillus* (44).. Furthermore, research has demonstrated that supplementation of conjugated linoleic acid can alleviate colitis by regulating cytokines and oxidative stress, maintaining the mucosal barrier, and rebalancing gut microbiota, including changes in *Bacteroides*, *Bifidobacterium*, and *Odoribacter* (57).

In ARDS, gut microbiota alterations affect the intestinal barrier and disease progression. Disrupted gut microbiota circadian rhythms in sepsis/ARDS patients are noted, but probiotics aid intestinal defense and immune response (58). COVID-19 impacts gut physiology, potentially causing dysbiosis and barrier damage, leading to immune and psychological issues (59). Chen et al. observed that lung inflammation in ARDS is exacerbated due to gut barrier dysfunction. They found that vancomycin could ameliorate this condition by altering the utilization of the gut microbial metabolite butyric acid (60). Tang et al. in an acute lung injury (ALI) animal model, suggested gut microbiota dysbiosis modulates the TLR4/NF-κB pathway in lungs, causing inflammation and stress, leading to lung injury (61). Zhou et al. found that in ALI, increased cytokines and lung microbiota changes cause gut dysbiosis and intestinal damage, exacerbating sepsis-induced ALI/ARDS (62).

### 3.3 The influence of gut microbiota on inflammatory response and immune cells in ARDS

In ARDS, an overactive inflammatory response causes immune system imbalance, worsening outcomes and prognosis. Disrupted intestinal barriers and gut microbiota changes can trigger local and systemic inflammation, promoting ARDS progression (63). In COVID-19, gut microenvironment balance significantly impacts systemic inflammation and ARDS onset (59). In a study conducted by Kyo et al., the lung microbiota in bronchoalveolar lavage fluid (BALF) from ARDS patients was examined. A significant correlation was found between the levels of *Staphylococcus, Streptococcus, Enterobacteriaceae* in the BALF and serum IL-6 levels (64). Another study revealed that antibiotics disrupt microbiota, altering host responses in LPS-induced lung inflammation and moderately increasing IL-6 in mice (65).

In fecal microbiota transplantation (FMT) treatments, probiotic mixtures containing bacteria such as *Bifidobacteria*, *Lactobacilli*, and *Thermophilic Streptococci* are commonly used (66). Additionally, ideal donors should have a high proportion of *Lachnospiraceae*, *Ruminococcaceae*, and *Clostridium scindens*, which are positively correlated with the secondary bile acids that inhibit Clostridium difficile infection spores (67). Integrating these specific microbial populations into FMT can enhance treatment effectiveness, especially in the context of increasing antimicrobial drug resistance. Li et al. demonstrated that in an ALI rat model, FMT not only reconstituted the gut microbiota structure and augmented bacterial gene abundance but also attenuated inflammatory cell infiltration and pulmonary exudation. Subsequent investigations revealed that FMT, by modulating the gut microbiota, could influence the TGF-β1/Smads/ERK signaling pathway, resulting in a reduction in the synthesis and secretion of inflammatory mediators *in vivo*. This, in turn, mitigated alveolar epithelial injury and ameliorated LPS-induced endotoxic ALI in the rats (68). Similarly, Tan et al. also indicated that FMT may affect the Keap1-Nrf2/ARE signaling pathway by modulating gut microbiota, enhancing the antioxidant stress response and reducing the production of oxidative damage-related products, thereby improving LPS-induced ALI in rats (69).

Lymphocytes, including T cells, Th cells, and Treg cells, play a pivotal role in mediating the inflammatory response in ARDS. Various studies have observed the distribution of T cells in ARDS. A recent investigation revealed that the Tregs/CD4^+^ percentage in the blood of ARDS patients was significantly higher compared to non-ARDS patients, while the Tregs/CD4^+^ percentage in the lung alveoli was lower (69). Similar research has also demonstrated significantly reduced CD4^+^, CD8^+^, and B lymphocyte counts in ARDS patients, along with activation of Th1 and Th2, inflammatory response cell apoptosis, cytotoxicity, and endothelial dysfunction (70, 71). Regarding gut microbiota, alterations in composition have been observed in ARDS patients, correlating with changes in lymphocyte counts, specifically CD3^+^ T cells, CD4^+^ T cells, and CD4^+^/CD8^+^ T cells (71). Nevertheless, further research is essential to gain a comprehensive understanding of this complex interaction.

### 3.4 Gut microbiota affects oxidative stress in ARDS

The activation of oxidative stress is intricately linked with the inflammatory response, leading to the increased production of pro-oxidants and the suppression of anti-oxidants (72, 73). In ARDS rat models, there is a marked promotion of oxidative stress, inflammatory response, cell apoptosis, and barrier disruption in alveolar epithelial cells (74), while LPS treatment significantly diminishes the activity of superoxide dismutase and glutathione peroxidase (75). Conversely, the inhibition of reactive oxygen species (ROS) and the enhancement of superoxide dismutase, catalase, and glutathione levels in lung tissue are considered beneficial in protecting animals from ARDS (68, 76). For ARDS patients, strategies aimed at reducing inflammation and oxidative stress can bolster the immune system, mitigating the severe impact of COVID-19 progression into ARDS, such approaches may also contribute to improving the treatment success and survival rates of COVID-19 patients (77, 78).

During the progression of ARDS, a complex network of interactions is observed between oxidative stress and alterations in gut microbiota. The imbalance in gut microbiota can trigger lung inflammation and oxidative stress, ultimately leading to gut-barrier-induced lung injury in animals with ALI (61). In a sequencing study utilizing fecal samples from 13 COVID-19 patients, Zhou et al. demonstrated that the metabolism of LPS, ketones, sphingolipids, and neutral amino acids was elevated, while the synthesis pathway of butyrate was anomalously diminished. These findings indicate that the gut microbiota of COVID-19 patients was in a state of oxidative stress (79).

### 3.5 Gut microbiota affects apoptosis in ARDS

Cellular apoptosis, a critical factor in the development of ARDS, has been found to contribute to the damage of the intestinal mucosal barrier. This damage, in turn, leads to subsequent alterations in gut microbiota, which can further exacerbate the condition. A study found that in LPS-induced ARDS models, inflammatory response, oxidative stress, cell apoptosis elevated, and barrier disruption in alveolar epithelial cells are promoted, while inhibition of cell apoptosis exerted a protective effect on ARDS, while use resveratrol could increase the abundance of *Lactobacillus casei*, which could be achieved by regulating the activation of the NLRP3 inflammasome and Rho GTPase axis, reducing the infiltration of inflammatory cells and the release of inflammatory factors, resveratrol helps to maintain the normal physiological function of the intestinal barrier, thereby reducing lung tissue damage and improving lung function (74). Studies have also suggested that in the BALB/c mouse model of ALI, the proportion of cell apoptosis was significantly increased, accompanied by an increase in the secretion of cytokines (80–82).

When alterations in gut microbiota occur during ARDS, these changes are typically associated with an increase in cell apoptosis within both lung and intestinal tissues. In a study involving staphylococcal enterotoxin B-induced ARDS in mice, researchers observed dysbiosis in both lung and gut microbiota, along with an increase in the proportion of cell apoptosis (83). Additionally, Xu et al. proposed that the administration of lysophosphatidylcholine could mitigate monocyte infiltration in the ARDS mouse model, an effect attributed to deoxynivalenol trichothecene mycotoxin galactosamine lipopolysaccharide bacteremia. This mitigation was achieved by modulating the MAPK/NF-κB signaling pathway and inhibiting monocyte apoptosis triggered by galactosamine lipopolysaccharide, subsequently enhancing survival rates and lung function (58).

### 3.6 The influence of gut microbiota on other cell functions (autophagy, pyroptosis, ferroptosis)

Cellular autophagy is often characterized as a ‘double-edged sword’ in various diseases and biological processes. It serves a protective role when functioning within normal limits but can become detrimental when overactivated (84, 85). The alteration of gut microbiota interacts with cellular autophagy, contributing to disease development. In research conducted by Cheng et al., it was discovered that in the piglet model treated with FMT, there was an increase in the abundance of beneficial microbes such as *Lactobacillus* and *Veillonella* and a decrease in *Enterobacteriaceae* and *Proteobacteria*. Furthermore, FMT also led to the production of short-chain fatty acids and other metabolic products. This activated AMPK, which in turn upregulated autophagy through pathways such as PGC-1α and mTOR. Additionally, FMT increased SIRT1 expression and promoted its deacetylation, activating PGC-1α and FOXO transcription factors. These changes enhanced mitochondrial biogenesis and the expression of autophagy-related genes, ultimately inducing autophagy (86). These findings indicate that FMT may enhance the composition of the gut microbiota, mitigate damage to the intestinal mucosal barrier, and diminish systemic inflammatory responses. This effect is achieved by modulating the metabolic function of the gut microbiota, particularly in the metabolism of linoleic acid. Cao et al. (87)demonstrated that berberine has the ability to inhibit the mammalian target of rapamycin complex 1 (mTORC1) axis. Such inhibition upregulates autophagy-related genes and proteins, such as Beclin-1, ATG5, and LC3II/I, in the intestinal epithelial cells of experimental animals, thereby inducing autophagy. Furthermore, berberine suppresses the TLR4/NF-κB signaling pathway, enhances the expression of tight junction proteins in the intestinal epithelial cells, reduces the phosphorylation of Claudin-2 and Occludin, and strengthens tight junctions and intestinal barrier function. It also stimulates the growth of beneficial bacteria like *Lactobacillus* and *Bifidobacterium* while inhibiting the growth of harmful bacteria such as *Escherichia coli*. This, in turn, increased the diversity and abundance of the gut microbiota in experimental animals. The study (87) also found that the use of 3-MA (an autophagy inhibitor) suppressed the expression of autophagy-related genes mentioned above, impeding the autophagy induction induced by berberine. Consequently, the anti-inflammatory and tissue-protective effects of berberine were weakened (87). Furthermore, a study conducted by Huang et al. confirmed that autophagy plays a vital role in enhancing the integrity of the intestinal mucosal barrier by mitigating oxidative stress in instances of acute severe pancreatitis (88). In the context of ARDS, studies have observed a significant suppression of autophagic activity in septic patients with this condition. The expression of autophagy-related markers such as LC3II, Beclin-1, RAB7, LAMP2, and p62 is markedly decreased. This finding indicates that the evaluation of autophagy-related proteins may hold diagnostic and prognostic significance for septic ARDS (89). Another study confirmed in an ARDS cell model and ALI rat model, treatment with penehyclidine hydrochloride enhanced proliferation and autophagy in ALI models, and reduced cell apoptosis and inflammation (90). However, how gut microbiota influence cell autophagy in ARDS now lacks adequate evidence.

Cell pyroptosis, a form of programmed cell death linked to inflammation, is mediated by inflammasomes like NLRP1, NLRP3, and AIM2, leading to caspase-1 activation and proinflammatory cytokine production, which results in excessive inflammation and cell death (91, 92). This process is crucial in ARDS, contributing to cytokine storms caused by various diseases (93, 94), and is evident in ARDS mouse models with increased NLRP3, IL-1β, and caspase-1 (95). Increased pyroptosis-related protein GSDMD-N in ARDS patients’ PBMCs suggests a role in exacerbating ARDS (96). However, the relationship between pyroptosis and gut microbiota in ARDS is less explored. Metabolite formononetin (FMN), a metabolite of *Parabacteroides* merdae, inhibits macrophage pyroptosis by interacting with NLRP3. Chen et al. found that reduced Parabacteroides merdae in pregnancy leads to less FMN, thereby facilitating pyroptosis and increasing septic responses and tissue damage (97). Understanding the link between cellular pyroptosis and gut microbiota alterations in ARDS is an emerging research area, potentially revealing new treatment approaches.

Ferroptosis, a newly recognized type of cell death characterized by iron-induced lipid peroxidation (98), is substantially activated in ARDS. This form of regulated cell death promotes inflammation and oxidative stress A parallel study has corroborated that ferroptosis plays a mediating role in inflammation within ALImouse models. By inhibiting ferroptosis, the expression of Nrf2 and HO-1 within the Keap1-Nrf2/HO-1 signaling pathway is augmented. This leads to the neutralization of hydroxyl radicals produced from excessive free iron, mitigating iron-induced cellular damage, and consequently exerting a protective effect against ALI (99). Furthermore, AUF1 plays a role in inhibiting the process of ferroptosis, thereby reducing sepsis-related ALI. This inhibition is achieved through the upregulation of the Nrf2-mediated antioxidant response and the downregulation of ATF3 expression, both of which are key mechanisms in controlling ferroptosis (100). In the contemporary landscape of medical research, the intricate interplay between ferroptosis and the gut microbiome in the context of ARDS remains a relatively uncharted domain. The exploration of how targeted modulation of the gut microbiome may influence ferroptosis, and consequently enhance the prognosis of ARDS, represents a promising avenue for future investigation. Such insights could herald innovative therapeutic strategies, offering new horizons in the management and treatment of ARDS.

## 4 The metabolic products of gut microbiota in ARDS

### 4.1 Short-chain fatty acids (butyric, acetic, and propionic acids) produced by gut microbiota in ARDS

The metabolic products of gut microbiota exert profound effects on the health and disease states of the host (human body). These metabolites include short-chain fatty acids (SCFAs), bile acids, amino acid metabolites, and vitamins, playing pivotal roles in maintaining intestinal health, regulating the immune system, and influencing systemic metabolic balance (101). SCFAs such as butyrate, propionate, and acetate, are products of the fermentation of cellulose by intestinal bacteria. They are crucial for the integrity of the intestinal mucosa, promoting the proliferation and repair of intestinal epithelial cells (102).

**Figure 2:**
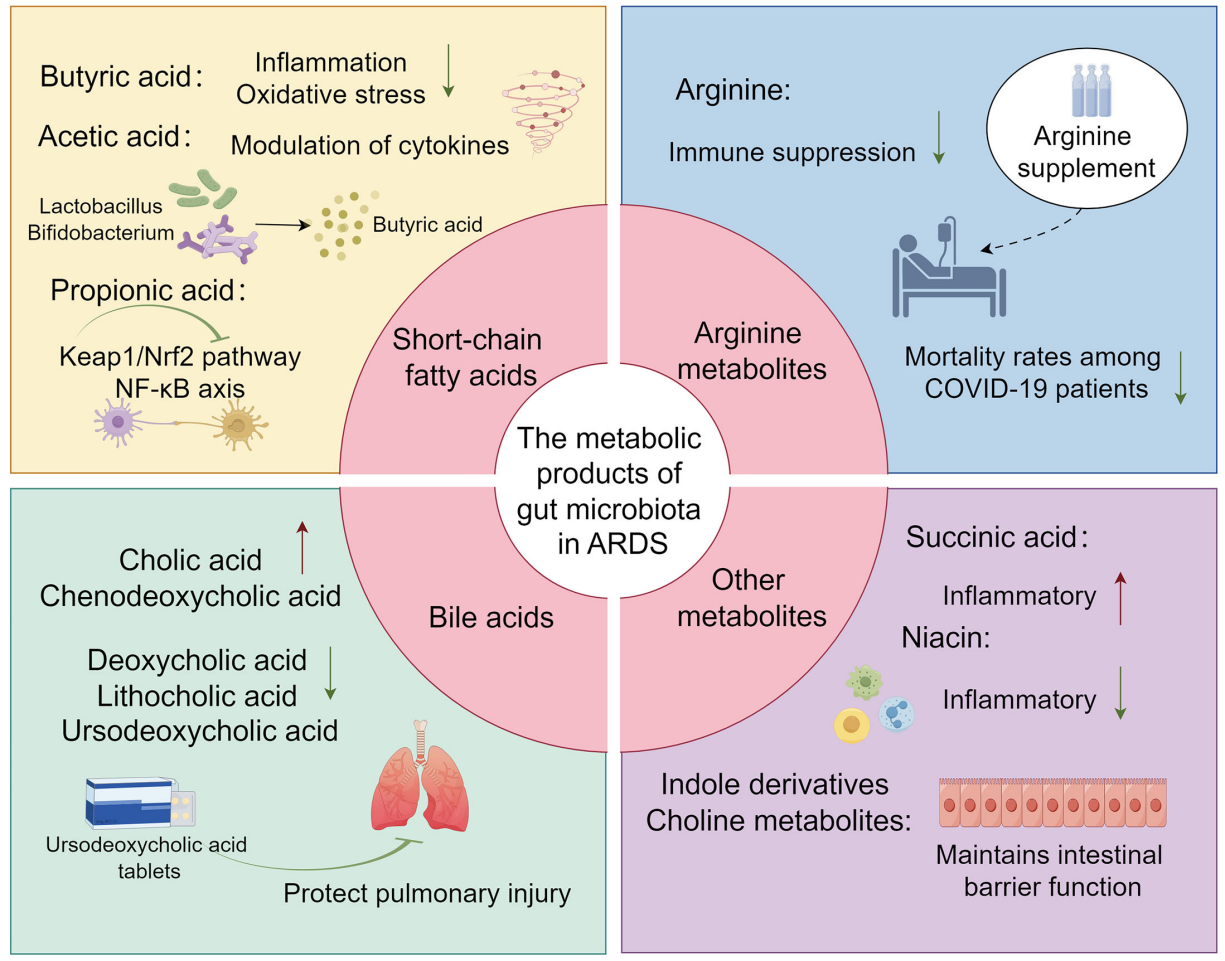
The overview image depicts several key metabolites produced by the gut microbiota, including short-chain fatty acids (such as Butyric, Acetic, and Propionic acids), Arginine, bile acids, and other potential metabolites (such as Succinic acid, Indole derivatives, Niacin, and Choline metabolites), and their roles in ARDS.

Butyric acid, a pivotal metabolite produced by gut microbiota, has been demonstrated to modulate the progression of ARDS in both clinical settings and *in vitro* or *in vivo* experimental studies. The regulation of this gut microbial metabolite by Vancomycin may attenuate the inflammatory response in ARDS, a mechanism potentially linked to the amelioration of gut barrier dysfunction in patients with ARDS (60). Another study identified a reduction in the synthesis of butyrate by gut microbiota in COVID-19 patients, indicative of an oxidative stress state potentially mediated through the dysfunction of butyrate metabolism (79). Subsequent research has revealed that butyrate may ameliorate inflammation and mitigate oxidative stress in ARDS caused by COVID-19. This suggests that butyrate could serve as an alternative to dexamethasone for treating severe infections, thereby potentially preventing the progression to severe ARDS and the resultant Multiple Organ Dysfunction Syndrome (MODS) (103). In cell and animal models, the gut microbiota metabolite butyrate promoted M2 macrophage polarization, facilitated goblet cell generation, and restored its mucosal barrier repair action *in vivo* and *in vitro (* 104). This also suggests that butyrate is involved in the regulation of epithelial barrier integrity. Studies also found in ARDS mice there was a significant increase in pro-inflammatory cytokines such as IFN-γ and IL-17, which might be associated with the dysbiosis of the lung and gut microbiome, with a significant reduction in the production of propionate, isobutyrate, and butyrate (83). Furthermore, the administration of anandamide (AEA) has been demonstrated to augment the abundance of beneficial gut bacteria, such as *Lactobacillus* and *Bifidobacterium*. This enhancement is also associated with an improvement in the overall architecture of the gut microbiota and an elevation in the concentration of short-chain fatty acids, particularly butyrate. AEA’s involvement in the regulation of the gut-lung axis, its mitigation of inflammatory responses, and its protective role in ARDS have been substantiated (105) (**Figure 2**) (**Table 2**).

**Table 2:** The metabolic products of gut microbiota in ARDS.

Literature Number	Types of metabolites	Metabolite name	Role in ARDS
(60, 79, 83), (103–105), (106–108), (109–111), (112–114), (115) (116, 117), (118) (119), (120–123, 106), (124–126), (127) (128, 129)	SCFA Arginine Bile Acid Other metabolites	Butyric Acid Acetic Acid Propionic Acid Arginine Arginine (via L-arginine-modified liposomes) Arginine (synthesized from Glutamine) Cholic acid, chenodeoxycholic acid Deoxycholic acid, Lithocholic acid, Ursodeoxycholic acid Succinic Acid Indole Derivatives Nicotinic acid Choline Metabolites	Reduces ARDS severity, decreases inflammation, promotes M2 macrophage polarization, improves gut and mucosal barrier function, and regulates immune responses. Uncertain role in ARDS, possibly affects inflammation in specific mouse models. Inhibits activation of NLRP3 inflammasomes, modulates Keap1/Nrf2 pathway, inhibits NF-κB axis activation. Improves inflammatory conditions. Direct correlation with MDSCs expansion in COVID-19, mitigates immune suppression, reduces duration of mechanical ventilation, decreases mortality rates. Used in curcumin treatment to target pulmonary M1 macrophages, modulates immunological responses in ALI/ARDS. Improve clinical outcomes in respiratory illnesses, therapeutic potential in ARDS treatment. Elevated levels of bile acids and involved in regulating bile acid metabolism. Produced by Clostridium with 7α-dehydroxylating capabilities, play a role in maintaining gastrointestinal microbiome homeostasis. Protect against pulmonary injury. Linked to protein succinylation, activation of SUCNR1, eliciting pro-inflammatory responses, potential therapeutic target in ischemia-reperfusion injuries. Regulatory influence on microbial behavior, host-pathogen interactions, enhances host epithelial barrier integrity, potential roles in ARDS. Agonist for GPR109A, suppresses NF-κB, anti-inflammatory and immunomodulatory properties, influence in ARDS still lacks evidence. Found in pulmonary surfactants, mitigates inflammation, anti-inflammatory properties, distinct metabolic profiles in influenza and COVID-19-induced ARDS.

Evidence of acetate production by gut microbiota in relation to ARDS is scant in recent studies. Some investigations have hinted at a correlation between the levels of BALF cytokines and specific gut microbiota metabolites, such as acetate and butyrate, in LPS-induced ALI mice (106–108). While these initial explorations have begun to shed light on the association between acetate and butyrate with ARDS, the exact role of acetate within ARDS remains elusive and inadequately defined. The prevailing research, largely confined to animal models and particular human cohorts, has limited the generalizability of these insights. Therefore, further comprehensive and nuanced studies are urgently needed to unravel the intricate mechanisms through which gut microbiota metabolites, specifically acetate and butyrate, modulate ARDS at the molecular and cellular levels.

During the progression of ARDS, the synthesis of propionic acid is posited to ameliorate inflammatory responses and mitigate pulmonary injury. Mohammed et al. conducted an assessment of alterations in lung inflammation, gut/lung microbiota, and SCFA production in ARDS mice. Their findings revealed that propionate possesses the capability to suppress the inflammatory response induced by ARDS (109). Another study elucidated that FMT modulates the structure and function of the gut microbiota, fostering the growth of health-associated functional microbiota, thereby enhancing the production of acetic and propionic acids. This contributes to the protective effect on mice with ARDS. Specifically, propionic acid, acting on the GPR43 receptor on macrophages, inhibits the activation of NLRP3 inflammasomes, suppressing the release and synthesis of TNF-α, IL-1β, and IL-6. This attenuation of the inflammatory response leads to a reduction in lung injury during ARDS (110). Additionally, Zhang et al. demonstrated through animal experiments that sodium propionate has the ability to modulate the Keap1/Nrf2 pathway and inhibit NF-κB axis activation. This leads to a reduction in the inflammatory response and oxidative stress in rats with ALI, thereby ameliorating lipopolysaccharide (LPS)-induced ALI (111).

Propionic acid exerts protective effects in ARDS through multiple targets, including tissue repair, antioxidant and anti-inflammatory effects, maintenance of barrier integrity, modulation of receptor-mediated signaling, and regulation of immune cell function. These mechanisms interact with each other to collectively alleviate the occurrence and progression of ARDS. However, additional studies are necessary to validate and expand upon these mechanisms, as well as to better understand their specific manifestations and contributions. This will provide a robust theoretical foundation for potential clinical applications. Butyrate, acetate, and propionate, as representative products of short-chain fatty acids produced by gut microbiota metabolism, provide new targets for ARDS treatment. However, clinical research and application of these three compounds are still lacking. Future research is warranted to explore their efficacy and safety. In comparison, the study of acetate is relatively limited compared to propionate and butyrate, with unclear mechanisms. Therefore, more attention needs to be paid to the differential study of its role and mechanisms in ARDS and the speed of clinical translation.

### 4.2 Arginine metabolites produced by gut microbiota in ARDS

Arginine is an essential amino acid in the human body, playing a vital role in various physiological processes (112). It serves as a substrate for the synthesis of nitric oxide, a critical signaling molecule involved in numerous aspects of vascular and immune functions. Arginine may influence patients’ immune nutrition and nitric oxide metabolism to improve patients’ inflammatory condition (113). It also has the ability to inhibit the expression of the T-cell receptor complex CD3, thereby affecting the proliferation and differentiation of T-cells (114, 130). A clinical investigation encompassing 26 patients with COVID-19 revealed a direct correlation between the expansion of myeloid-derived suppressor cells (MDSCs) in association with COVID-19 and a reduction in lymphocytes, coupled with increased arginase activity. Furthermore, supplementation with arginine was observed to mitigate immune suppression, reduce the duration of mechanical ventilation, and decrease mortality rates among the patients (131). An additional study corroborated that curcumin confers protective effects in a rat model of ALI/ARDS by specifically targeting pulmonary M1 macrophages through the use of L-arginine-modified liposomes, thereby modulating immunological responses (115), playing an immunomodulatory role.

Glutamine acts as an essential precursor in the biosynthesis of arginine. Arginine can be synthesized from glutamine either through the enzymatic catalysis of glutamate dehydrogenase or via the conversion of ornithine derived from glutamine, facilitated by glutamine-ornithine transcarbamylase. While supplementation with both glutamine and arginine has demonstrated efficacy in improving clinical outcomes for patients with respiratory illnesses, the therapeutic potential of this approach in the treatment of ARDS warrants further comprehensive studies and rigorous scientific investigation (116, 117).

### 4.3 Bile acid synthesized by gut microbiota in ARDS

Bile acids are a group of complex molecules derived from cholesterol, primarily synthesized in the liver and secreted into the small intestine (132). They play a crucial role in the digestion and absorption of lipids and fat-soluble vitamins. Beyond their role in digestion, bile acids are increasingly recognized as signaling molecules that regulate metabolic processes within the host. The composition of the gut microbiota is modulated by bile acids; reciprocally, the microbiota exerts a regulatory function in bile acid metabolism (133). Both *Clostridium scindens* and *Clostridium sordellii* are gut bacteria with 7α-dehydroxylating capabilities, facilitating the biotransformation of primary bile acids into secondary bile acids. These bacterial species additionally secrete specific tryptophan-derived antibiotics, identified as 1-acetyl-β-carboline and turbomycin A. These antibiotics act by inhibiting the formation of the septum during bacterial cell division, thereby exerting a suppressive effect on the growth of *Clostridium difficile* as well as other constituents of the gut microbiota. This regulatory interaction plays a pivotal role in maintaining the homeostasis of the gastrointestinal microbiome (119). In patients with ARDS, existing evidence suggests a dysregulation in bile acid metabolism, characterized by elevated levels of primary bile acids such as cholic acid and chenodeoxycholic acid, and a concomitant reduction in secondary bile acids, including deoxycholic acid, lithocholic acid, and ursodeoxycholic acid. This distinct profile of plasma bile acid concentrations may function as an adaptive physiological response in the pathogenesis of ARDS (118). The research conducted by Niu F and colleagues demonstrated that ursodeoxycholic acid exerts a protective effect against pulmonary injury resulting from ARDS. In a clinical investigation conducted by Harnisch and colleagues, it was observed that patients with ARDS exhibited elevated levels of tauro-conjugated bile acids and a significantly diminished glycine-to-taurine conjugation ratio (G/T ratio) in comparison to the control cohort (134). In a study led by Kean and associates, it was elucidated that pediatric patients in critical condition, encompassing those diagnosed with ARDS, manifest gut microbiota dysbiosis. This imbalance is typified by a reduction in the genus *Bifidobacterium* and an augmentation in the genus *Veillonella*. Concurrently, this dysbiosis is associated with perturbations in bile acid metabolism, including a decline in dehydroxy bile acids and hydrogen ions, coupled with an elevation in hydroxylated hydrophobic bile acids (135).

The current body of research elucidates the putative roles of gut microbiota and bile acid metabolism in the pathophysiology of ARDS. These findings not only offer potential biomarkers for the diagnosis and therapeutic intervention of ARDS but also lay the groundwork for subsequent investigations. Future research endeavors should focus on delineating the precise mechanistic contributions of gut microbiota and bile acid metabolic pathways in the etiology of ARDS, thereby paving the way for the exploration of targeted modulation of these systems for prevention or treatment of the condition.

### 4.4 Potential roles of other metabolites (succinic acid, indole derivatives, niacin, and choline metabolites) produced by gut microbiota in ARDS

The occurrence of membrane-bound succinates is intricately linked with protein succinylation, a specific form of post-translational modification. This biochemical modification plays a pivotal role in the stabilization of hypoxia-inducible factortranscription factors, the initiation of pro-inflammatory signaling cascades, epigenetic modulation, and the production of reactive ROS (120–122). During episodes of intestinal ischemia/reperfusion (I/R), there is a marked elevation in plasma succinate concentrations. This increase functionally activates the succinate receptor 1 (SUCNR1) expressed on alveolar macrophages. Activation of SUCNR1 subsequently initiates the Phosphoinositide 3-Kinase-Protein Kinase B (PI3K-AKT)/Hypoxia-Inducible Factor-1αintracellular signaling cascade. This molecular eventuality culminates in the elicitation of pro-inflammatory responses within alveolar macrophages, thereby serving as a pathophysiological precursor to acute lung injury in the context of intestinal I/R (106, 123). According to the research conducted by Chouchani et al., succinate serves as a pivotal modulator in the induction of mitochondrial reactive ROS during the reperfusion phase subsequent to ischemic events. This accumulation of succinate is notably ascribed to the reversal of enzymatic activity of succinate dehydrogenase during the ischemic phase (136). The aforementioned study elucidates that the inhibition of succinate accumulation and the attenuation of mitochondrial ROS generation serve as potential therapeutic targets for diseases induced by ischemia-reperfusion injuries and related pathological conditions. In the presence of high-risk factors for ARDS or during the onset and progression of ARDS, the precise role of succinate warrants further comprehensive investigation for a more detailed understanding.

Indole, a pivotal metabolite in bacterial tryptophan metabolism, exerts a multifaceted regulatory influence on microbial behavior and host-pathogen interactions. Specifically, indole modulates bacterial motility, biofilm formation, antibiotic resistance, and exerts multifunctional effects on host cell invasion and the expression of virulence factors. Upon exposure to indole, normal cells are capable of enhancing the integrity of the host epithelial barrier by upregulating the transcriptional activity of genes associated with epithelial barrier function (124). Additionally, indole can regulate cytokines to improve inflammation and damage (62). Recent research indicated a significant reduction in the levels of indole-3-acetate and indole-3-propionate in both septic mice and patients (125, 126). Evidence suggested that in chronic lung disease, metabolites produced by the gut microbiota exhibited anti-inflammatory and anti-infective activity, and during this progress, indole compounds and their derivatives have been shown to activate the aryl hydrocarbon receptor signaling in astrocytes, leading to the inhibition of inflammation in the central nervous system (137–139). These results can imply potential roles of indole and the derivatives in ARDS, while both clinical and experimental works should be done to confirm it. Additionally, Nicotinic acid, a gut microbiota-derived short-chain fatty acid, serves as an agonist for G protein-coupled receptor 109A (GPR109A) expressed on colonic macrophages and dendritic cells. Activation of GPR109A leads to the suppression of NF-κB signaling pathways and a concomitant reduction in proinflammatory cytokine synthesis. Simultaneously, it enhances the generation of regulatory T cells and IL-10, thereby manifesting both anti-inflammatory and immunomodulatory properties. These effects hold potential for mitigating gut microbiota dysbiosis and immune-mediated inflammation commonly observed in COVID-19 patients (127). In ARDS, the metabolism and influence of niacin still lack evidence.

Choline is ubiquitously present in all cell membranes and various secretions, predominantly in the form of phosphatidylcholine and sphingomyelin, particularly in pulmonary tissues. In the lungs, it is found in pulmonary surfactants and alveolar lipoproteins. Certain probiotics in the gut microbiota, such as *Bifidobacterium*, are capable of producing choline. Beyond maintaining intestinal barrier function, choline also mitigates inflammation induced by bacterial translocation and exhibits anti-inflammatory properties. A widespread deficiency of choline is observed in patients with cystic fibrosis, with a reduction in choline-producing probiotics in the gut microbiota being one of the primary contributing factors (129). A recent report employing metabolomic analysis has elucidated distinct metabolic profiles between influenza-induced ARDS and COVID-19-induced ARDS. This finding serves as a foundational basis for investigating the influence of gut microbiota-derived metabolites on the pathophysiology of ARDS (128). Further investigation into the anti-inflammatory properties of choline and its regulatory effects on gut microbiota, along with the exploration of niacin’s impact on the gut-lung axis, will constitute focal points in future research within the realm of ARDS.

## 5 Conclusion and prospect

The intricate crosstalk between gut microbiota and ARDS exerts a synergistic influence on the trajectory of disease progression. ARDS instigates perturbations in the compositional architecture of the gut microbiota, subsequently precipitating disruptions in the integrity of the intestinal mucosal barrier, bacterial translocation, endotoxin absorption, and eliciting anomalies in humoral immunity. These disruptions further manifest as dysregulated inflammatory responses, heightened oxidative stress, and cellular processes including apoptosis, autophagy, pyroptosis, and ferroptosis. Conversely, specific microbial metabolites, such as butyrate, propionate, arginine, and bile acids, have demonstrated therapeutic efficacy in attenuating the inflammatory and oxidative sequelae associated with ARDS. Recent advancements in the field have earmarked gut microbiota modulation as a novel therapeutic paradigm for ARDS management. Interventions designed to re-establish gut microbial homeostasis—through probiotic supplementation, provision of probiotic-enriched dietary sources, and targeted inhibition of pathogenic bacterial proliferation—have shown promise in fortifying the intestinal barrier, mitigating inflammatory cascades, and improving the clinical prognosis of ARDS patients. Despite these advancements, the current evidence base remains inadequate for a comprehensive understanding of the multifaceted roles that gut microbiota play in ARDS, especially concerning the mechanistic impact of distinct metabolites and the molecular pathways implicated in this complex interplay.

To comprehensively elucidate the intricate roles of gut microbiota and their metabolic byproducts in the pathophysiology of ARDS, a series of methodologically rigorous empirical studies are urgently warranted. Initially, an exhaustive investigation into the multifaceted roles and interdependent mechanisms of specific microbial metabolites within the ARDS pathophysiological milieu is imperative. Concurrently, further scrutiny is required to understand the intracellular signaling cascades, receptor-ligand interactions, and cytokine-mediated regulatory pathways between the gut microbiota and ARDS. Subsequently, a systematic categorization and characterization of the gut microbiota across diverse ARDS patient cohorts should be undertaken. This would facilitate the identification of specific microbial taxa significantly correlated with clinical biomarkers, thereby laying the groundwork for microbiota-centric, precision medicine-based therapeutic interventions. Such interventions may encompass the application of FMT and the formulation of individualized probiotic regimens. In the realm of clinical research, a multidimensional approach incorporating metagenomics, metabolomics, transcriptomics, and epidemiological studies of the gut microbiota is advocated. The focus of therapeutic strategies should transition from conventional symptomatic treatments, such as restrictive ventilation and prone positioning, to more targeted interventions aimed at restoring immunological homeostasis, mitigating inflammatory cascade amplification, and re-establishing the ecological stability of the gut-lung axis. Modulating the gut microbiota and fortifying the integrity of the intestinal barrier may serve as pivotal mechanisms for minimizing bacterial translocation, reducing endotoxin exposure, and maintaining systemic immunological balance, thereby potentially decreasing the incidence and severity of ARDS. Consequently, further and comprehensive research endeavors are anticipated to provide groundbreaking insights into the early diagnosis, targeted therapeutic interventions, disease trajectory, prognostic evaluation, and rehabilitation strategies for ARDS.

## Author contributions

D-WZ: Investigation, Project administration, Resources, Software, Supervision, Writing – review & editing. J-LL: Methodology, Software, Validation, Visualization, Writing – original draft. B-YD: Methodology, Project administration, Software, Visualization, Writing – original draft. M-YF: Conceptualization, Project administration, Writing – review & editing. XX: Formal Analysis, Methodology, Writing – review & editing. X-JQ: Funding acquisition, Methodology, Project administration, Writing – original draft. X-MF: Conceptualization, Methodology, Project administration, Resources, Writing – review & editing.
